# Risk factors for vertebral fracture in rheumatoid arthritis patients using biological disease-modifying anti-rheumatic drugs (cases over 5 years): An observational study

**DOI:** 10.1097/MD.0000000000038740

**Published:** 2024-07-05

**Authors:** Hiroto Tokumoto, Hiroyuki Tominaga, Shingo Maeda, Hiromi Sasaki, Ichiro Kawamura, Takao Setoguchi, Noboru Taniguchi

**Affiliations:** a Department of Orthopaedic Surgery, Graduate School of Medical and Dental Sciences, Kagoshima University, Kagoshima, Japan; b Department of Bone and Joint Medicine, Graduate School of Medical and Dental Sciences, Kagoshima University, Kagoshima, Japan; c Department of Orthopaedic Surgery, Japanese Red Cross Kagoshima Hospital, Kagoshima, Japan.

**Keywords:** anti-rheumatic drugs, biological disease-modifying, modified Health Assessment Questionnaire, osteoporosis, rheumatoid arthritis, vertebral fracture

## Abstract

While biological disease-modifying anti-rheumatic drugs (bDMARDs) are considered beneficial for preventing osteoporosis and bone fracture, it is unclear whether bone loss is involved in the development of vertebral fracture, and few reports have examined the factors related to vertebral fracture in rheumatoid arthritis (RA) patients using bDMARDs. This study aims to identify factors influencing vertebral fracture in RA patients treated with bDMARDs. We retrospectively examined the records of 129 RA patients treated with bDMARDs for over 5 years. The lumbar spine and femoral bone mineral density, Disease Activity Score-28-C-Reactive Protein (DAS28-CRP) value, Simplified Disease Activity Index (SDAI), and modified Health Assessment Questionnaire (mHAQ) score were evaluated. The frequency of new vertebral fracture during the study and their risk factors were investigated. A comparison between the fracture group and the nonfracture group was performed. Multivariate analysis was performed using logistic regression analysis to detect risk factors for new vertebral fracture. The number of patients with new vertebral fracture during follow-up was 15 (11.6%) of the 129 patients in the study. Age and mHAQ score were significantly higher and lumbar spine and femoral neck bone mineral density were significantly lower in the fracture group than the nonfracture group. The risk factors for new vertebral fracture during the disease course were older age and higher mHAQ score indicating no remission over the 5 years of follow-up. In this study, there was no significant difference in disease indices such as the DAS28-CRP value and the SDAI between the fracture and nonfracture groups, suggesting an effective control of RA with bDMARDs. However, age and the mHAQ score, an index of RA dysfunction, were significantly higher in the fracture group. These results suggest that improving functional impairment may be important to prevent vertebral fracture in patients using bDMARDs.

## 1. Introduction

Rheumatoid arthritis (RA) is a chronic, systemic inflammatory disorder characterized by joint inflammation and destruction. Recent advances have broadened the spectrum of treatment options for RA, notably with the introduction of biological disease-modifying anti-rheumatic drugs (bDMARDs). Treatment with bDMARDs not only induces rapid remission but also decreases radiological progression and disability.^[1]^

There is emerging evidence suggesting that biologics might positively influence bone metabolism and bone remodeling.^[2]^ Achieving clinical and structural remission, as well as functional remission, is the primary goal of RA treatment. The modified Health Assessment Questionnaire (mHAQ, with a remission criterion of ≤0.5) serves as a widely used tool for evaluating this aspect.^[3]^ The mHAQ, which comprises 8 questions, provides insights comparable to those obtained from the 20 questions in the HAQ.^[1]^ Both HAQ and mHAQ are effective for screening RA patients for physical function.^[2]^

Studies have shown that RA patients treated with bDMARDs experience femoral bone loss, which is associated with higher steroid use and shorter disease duration.^[4]^ Additionally, risk factors for having a femoral neck bone mineral density (BMD) below 70% of the young adult mean (YAM) include a low Geriatric Nutritional Risk Index (GNRI) as a nutritional marker and female sex.^[5]^ However, the role of bone loss in vertebral fractures among RA patients treated with bDMARDs remains unclear, with limited research on the risk factors for vertebral fracture development in this group.

This study aimed to evaluate risk factors for vertebral fracture in RA patients treated with bDMARDs.

## 2. Materials and methods

This research has been approved by the IRB of the authors’ affiliated institutions. This research was executed in accordance with the Helsinki Declaration of 1975, as revised in 2008. All subjects gave their informed consent for inclusion before participating in the study.

### 2.1. Participants

We retrospectively examined the medical records of the patients diagnosed with RA according to the 2010 American College of Rheumatology criteria. These patients were treated at the authors’ affiliated institutions with bDMARDs, including tumor necrosis factor (TNF)-α inhibitors (e.g., infliximab, adalimumab), interleukin-6 inhibitors (e.g., tocilizumab), and cytotoxic T lymphocyte-associated molecule-4 (CTLA-4)-Ig (i.e., abatacept).

### 2.2. Demographic and disease-related data

Demographic and clinical data were collected from the medical records of 1178 patients with RA who were followed up for more than 5 years at our institution between 2011 and 2014. Overall, 376 of the 1178 patients were treated with bDMARDs. These data included several key parameters: sex, age, disease duration, and estimated glomerular filtration rate (eGFR) (mL/min/1.73 m^2^). Additionally, we noted the average prescribed dose of methylprednisolone and the GNRI. For assessing RA disease activity, we used both the Disease Activity Score-28-C-Reactive Protein (DAS28-CRP), and the Simplified Disease Activity Index (SDAI). The mHAQ score was used to measure physical function. Finally, we collected data on serum CRP levels (mg/dL) and BMD at the lumbar spine and femoral neck (g/cm^2^). After 1 year, assessments of bone mass and nutrition were repeated to evaluate for improvements.

After 5 years, any vertebral fractures were evaluated by standing thoracolumbar spine radiographs, and new vertebral fractures were noted. Furthermore, the mHAQ was re-administered. Vertebral fracture was defined as a grade 1 or higher fracture according to the semiquantitative (SQ) grading method as performed by a spine surgeon accredited by the Japanese Society for Spine Surgery and Related Research and an orthopedic specialist. The fracture group was defined as those patients with new vertebral fractures during follow-up, and the nonfracture group was defined as those patients without new vertebral fractures. In the fracture group, patients were prescribed corsets, but none underwent surgical intervention.

### 2.3. Dual-energy X-ray absorptiometry measurements

BMD was examined using a Discovery DXA system (Hologic, Waltham, MA, USA) between December 2011 and December 2014. The Japanese Society for Bone and Mineral Research recommends diagnosing of primary osteoporosis when BMD, expressed as a percentage of the YAM, falls below 70%. All patients diagnosed with osteoporosis received treatment for osteoporosis. One year following the initial staging, we reexamined the BMD in the lumbar spine and femoral neck.

### 2.4. Statistical analysis

Parameters were compared with the Wilcoxon test, Fisher exact test, and multivariate logistic regression analysis to determine risk factors for vertebral fracture. A stepwise procedure was used with the inclusion of variables that had a significance level of *P* < .05. The bivariate correlation analysis was performed for the correlation of variables. *P* < .05 was considered statistically significant. The JMP software program (version 16; SAS Institute, Cary, NC, USA) was used for statistical analysis.

## 3. Results

### 3.1. Patients’ demographic and clinical characteristics

**Figure 1.**
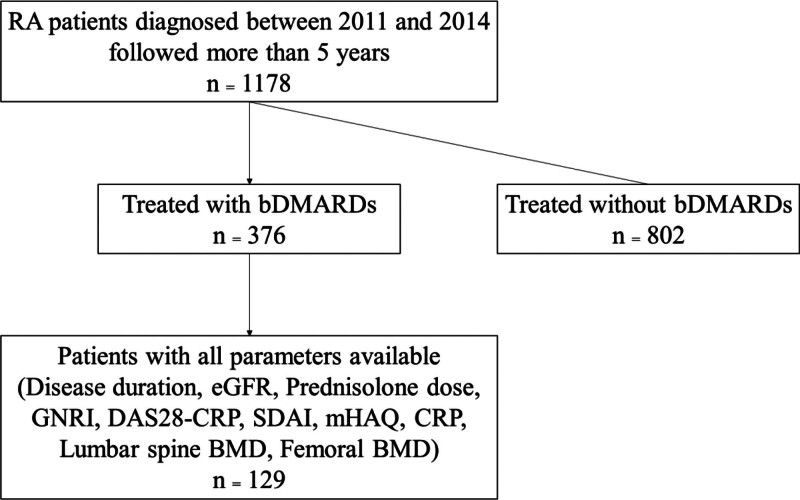
Flow chart of this study. RA = rheumatoid arthritis, bDMARDs = biological disease-modifying anti-rheumatic drugs, eGFR = estimated glomerular filtration rate, GNRI = geriatric nutritional risk index, DAS28-CRP = Disease Activity Score-28-CRP, SDAI = Simplified Disease Activity Index, mHAQ = Modified Health Assessment Questionnaire, CRP = serum C-reactive protein concentration, BMD = bone mineral density.

The flowchart of this study is shown in Figure 1. The demographic and clinical characteristics of the 129 patients who underwent DXA scanning of the femoral neck and lumbar spine are shown in Table 1.

**Table 1:** Patient demographic data.

Variable	Value
Female, ratio	109/129 (84.5%)
Age (yr)	60 (54–66)
Disease duration (yr)	10.0 (5.0–17.0)
eGFR (mL/min/1.73 m^2^)	76.0 (65.2–85.3)
Prednisolone dose (mg)	0.0 (0.0–2.0)
GNRI	105.9 (102.0–108.9)
DAS28-CRP	2.5 (1.6–3.3)
SDAI	5.8 (2.6–12.9)
mHAQ	0.5 (0.0–1.3)
CRP (mg/dL)	0.08 (0.03–0.28)
Lumbar spine BMD (g/cm^2^)	0.88 (0.77–0.98)
Lumbar spine YAM (%)	86.0 (76.0–97.0)
Lumbar spine BMD ≤ 70% of YAM	13 (10.1%)
Femoral BMD (g/cm^2^)	0.61 (0.52–0.68)
Femoral YAM (%)	76.0 (66.0–84.5)
Femoral BMD ≤ 70% of YAM	49 (38.0%)
Old vertebral fracture (no.)	19 (14.7%)

BMD = bone mineral density, CRP = serum C-reactive protein concentration, DAS28-CRP = Disease Activity Score-28-CRP, eGFR = estimated glomerular filtration rate, GNRI = geriatric nutritional risk index, MHAQ = Modified Health Assessment Questionnaire, SDAI = Simplified Disease Activity Index, YAM = young adult mean.

Among the 129 patients, approximately 85% were female, with a median age was 60 years. The lumbar and femoral percentages of the YAM were 86% and 76%, respectively, and the GNRI was 105.9, indicating good nutritional status. The DAS28-CRP value of the patients indicated remission, and the SDAI showed low activity (Table 1).

### 3.2. Comparison of demographic and clinical characteristics between the fracture and nonfracture groups

A total of 129 patients were on biologics for RA and were followed up for more than 5 years. New vertebral fractures occurred in 15 patients (11.6%) during the 5 years of RA treatment, with 10 of these fractures located in the thoracolumbar transition region. About 1 patient had SQ grade 1, 9 had SQ grade 2, and 5 had SQ grade 3 fractures. Comparative analysis revealed that the new vertebral fracture group had significantly higher ages and mHAQ scores, along with lower BMD in the femur and lumbar spine (Table 2). There were negative correlations between the BMD and YAM in the femoral neck and the disease duration (Supplemental Table 1, http://links.lww.com/MD/N68). There was no correlation between age and the mHAQ score, and there was no difference in the incidence of new vertebral fractures between types of bDMARDs. Old vertebral fractures were not a risk factor for new vertebral fractures. In the nonfracture group, the mHAQ score indicated that the patients remained in remission over the 5 years of follow-up (Table 3).

**Table 2:** Comparison of baseline data between fracture and nonfracture groups.

Vertebral fracture	− (N = 114)	+ (N = 15)	*P*
Female (n, %)	95 (83.3%)	14 (93.3%)	0.46
Age (yr)	59 (53–65.2)	65 (63–75)	0.001*
Disease duration (yr)	10 (5–16)	15 (7–20)	0.24
eGFR (mL/min/1.73 m^2^)	76.6 (67.1–85.2)	68.7 (61.6–96.2)	0.75
Corticosteroid (n, %)	47 (41.2%)	7 (46.7%)	0.78
Femur BMD (g/cm^2^)	0.62 (0.55–0.69)	0.52 (0.49–0.58)	0.003*
Lumbar spine BMD (g/cm^2^)	0.88 (0.77–0.99)	0.83 (0.72–0.88)	0.04*
GNRI	106.1 (102.5–109.1)	103.6 (99.7–107.5)	0.12
DAS28-CRP	2.5 (1.6–3.3)	2.5 (1.3–3.0)	0.39
SDAI	5.9 (2.5–13.5)	4.3 (3.1–11.6)	0.55
mHAQ score at start of observation	0.25 (0–1.0)	0.9 (0.3–1.4)	0.04*

BMD = bone mineral density, DAS28-CRP = Disease Activity Score-28-CRP, eGFR = estimated glomerular filtration rate, GNRI = geriatric nutritional risk index, MHAQ = Modified Health Assessment Questionnaire, SDAI = Simplified Disease Activity Index.

* Statistically significant.

**Table 3:** Involvement of new vertebral fractures with types of bDMARDs and patient parameters.

Vertebral fracture	− (N = 114)	+ (N = 15)	*P*
TNF-α inhibitors (infliximab, adalimumab) (n, %)	47 (41.2%)	8 (53.3%)	0.41
Tocilizumab (n, %)	39 (34.2%)	3 (20.0%)	0.38
Abatacept (n, %)	28 (24.6%)	4 (26.7%)	0.99
bDMARD switch (n, %)	12 (10.5%)	1 (6.7%)	0.99
GNRI improvement (at 1 yr) (n, %)	49 (43.0%)	7 (46.7%)	0.99
Lumbar spine BMD improvement (at 1 yr) (n, %)	65 (57.0%)	10 (66.7%)	0.58
Femur BMD improvement (at 1 yr) (n, %)	50 (43.9%)	7 (46.7%)	0.99
Old vertebral fracture (n, %)	17 (14.9%)	2 (13.3%)	0.99
mHAQ score maintained for 5 yr (n, %)	72 (61.4%)	4 (26.7%)	0.01*

bDMARD = biological disease-modifying anti-rheumatic drug, BMD = bone mineral density, GNRI = geriatric nutritional risk index, MHAQ = Modified Health Assessment Questionnaire.

* Statistically significant.

### 3.3. Risk factors for new vertebral fracture in RA patients

Multivariate logistic regression analysis showed that the risk factors for new vertebral fracture during the disease course were older age and an mHAQ score indicating no remission over the 5 years (Table 4).

**Table 4:** Multivariate logistic regression analysis of risk factors related to new vertebral fracture.

Factors for new vertebral fractures (5 yr)	OR (95% CI)	*P*
Age	1.11 (1.03–1.20)	0.01*
mHAQ score maintained for 5 yr	0.27 (0.08–0.94)	0.04*

CI = confidence interval, MHAQ = Modified Health Assessment Questionnaire, OR = Odds ratio.

* Statistically significant.

## 4. Discussion

Limited research has examined the risk factors for vertebral fracture in RA patients treated with bDMARDs. This study aimed to identify such risk factors. We observed that new vertebral fractures were more likely to occur in older patients and less likely in those whose mHAQ score suggested remission. Notably, a high mHAQ score emerged as a significant risk factor for vertebral fractures in RA patients under bDMARDs therapy, even in cases of low RA activity.

The median DAS28-CRP value in RA patients treated with bDMARDs was 2.5, and the SDAI was 5.8, indicating low disease activity. Despite remaining in a low disease activity status, 11.6% of these patients showed a BMD ≤ 70% YAM in the lumbar spine. El Maghraoui et al reported that the prevalence of vertebral fracture was increased in patients with higher DAS28 scores.^[6]^ RA is caused by inflammatory cytokines that lead to bone destruction and loss of bone mass.^[7]^ Recent studies have shown that RA can be alleviated by bDMARDs. bDMARDs significantly improve RA symptoms, function, and remission rates in patients with prior treatment failure with methotrexate or conventional disease-modifying anti-rheumatic drugs (cDMARDs).^[8]^ Van der Heijde et al^[9]^ found that the use of bDMARDs led to improved physical function in patients with RA. Notably, low physical ability is associated with an increased risk of vertebral fracture.^[10]^ In this study, bDMARDs resulted in remission based on the DAS28 value and low activity according to the SDAI, and the mHAQ score was high in the vertebral fracture group.

It has been reported that the mHAQ score is substantially higher in RA patients with new vertebral fractures.^[11]^ Therefore, it is essential to prevent vertebral fractures to maintain the functional status of RA patients.^[11]^ However, the report by Omata et al does not mention the type of RA medication used to achieve this.^[11]^ In this study, in which bDMARDs were used by all patients, vertebral fractures occurred in 15 (11.6%) who were almost asymptomatic, averaging 1.3 mg/d prednisolone, suggesting that bDMARDs controlled their symptoms. Another group also showed that an mHAQ score >1.5 is correlated with bone loss in the proximal femur.^[12]^ In this study, the median mHAQ score was 0.5; an mHAQ score of 0.5 or less is considered to indicate functional remission,^[3]^ suggesting that low activities of daily living (ADLs) are likely to cause fracture even if RA activity is suppressed.

Dirven et al^[13]^ reported that disease activity over time was higher in patients with vertebral fracture. This may be because of high levels of pro-inflammatory cytokines such as TNF-α and IL-6 in patients with high RA activity. Ghazi et al reported that the prevalence of vertebral fracture was inversely related to the use of steroids,^[14]^ and Dirven et al^[13]^ claimed no association between vertebral fracture and steroid use. In this study, steroids were not a risk factor for vertebral fracture because RA was controlled well. Takahashi et al found that the class and duration of biologics therapy were not significantly different in the BMD < 70% of femoral YAM and BMD ≥ 70% of femoral YAM groups.^[15]^

In addition to influencing disease activity, biologics may prevent bone loss via a direct effect on bone metabolism. It is well recognized that TNF-α induces differentiation of osteoclast precursors through synergistic action with RANKL.^[16]^ Shin et al did not find a significant difference in the risk of fracture between TNF initiators and abatacept or tocilizumab among RA patients.^[17]^ Our data showed no significant differences among the different bDMARDs in the incidence of vertebral fracture in RA patients.

It has been reported that risk factors for the least significant lumbar spine and femoral neck reduction were high-dose methylprednisolone use and short disease duration, respectively.^[4]^ However, another study reported that a longer RA disease duration is significantly related to the loss of BMD in the femoral neck and total femur.^[18]^ Our findings showed negative correlations between the BMD and YAM in the femoral neck and the disease duration. A recent study has suggested that bDMARDs have a protective effect on bone loss compared to cDMARDs.^[19]^ On the other hand, another report did not show any significant difference in vertebral fractures between bDMARD users and non-users.^[20]^ In RA patients with relatively controlled inflammation, considerations other than bone loss may be necessary to prevent vertebral fractures.

The low GNRI, used here as a nutritional index, was a risk factor for bone loss at the femoral neck in bDMARD patients. Complementary nutritional therapies might improve RA activity and osteoporosis in RA patients who have undergone treatment with bDMARDs.^[5]^ In this study, we tracked changes in the GNRI and bone mass; however, there was no association between improved nutrition and bone mass (femoral neck) and new vertebral fractures, suggesting that nutritional status may be an indicator of RA activity.

Patients who have had RA for a longer duration may be more likely to experience remission of the inflammatory state. Some people have a high mHAQ score even when their RA disease is in remission. It has been reported that abdominal trunk muscle weakness is a significant risk factor associated with osteoporotic vertebral fracture in the lower thoracic or lumbar spine.^[21]^ Among RA patients who achieve disease control with bDMARDs, vertebral fractures may be prevented by maintaining ADLs through rehabilitation and other means.

Our study has several limitations. First, being a single-center cohort study, it may have been subjected to selection bias. Future research should involve a multicenter approach to confirm our findings. Second, we were unable to assess the dietary habits of patients in their home environments. Third, our study also did not incorporate nutritional interventions. Future studies should aim for a more comprehensive determination of variables and patient samples to accurately identify crucial risk factors. Fourth, there is a possibility that we missed patients with RA who were followed up for more than 5 years but may have transferred to other hospitals or dropped out of the study.

## 5. Conclusion

In patients with RA using bDMARDs, age and the mHAQ score, an index of RA dysfunction, were significantly higher in the fracture group despite the effectiveness of bDMARDs in controlling RA. These results suggest that improving functional impairment may be important to prevent vertebral fracture in patients using bDMARDs.

## Author contributions

**Conceptualization:** Hiroto Tokumoto, Hiroyuki Tominaga, Takao Setoguchi.

**Data curation:** Hiroto Tokumoto, Hiromi Sasaki, Ichiro Kawamura.

**Formal analysis:** Hiroto Tokumoto, Hiroyuki Tominaga.

**Writing – original draft:** Hiroto Tokumoto, Hiroyuki Tominaga.

**Supervision:** Hiroyuki Tominaga, Shingo Maeda, Noboru Taniguchi.

**Writing – review & editing:** Shingo Maeda, Noboru Taniguchi.

**Validation:** Hiromi Sasaki, Ichiro Kawamura.

**Investigation:** Takao Setoguchi.

## Supplementary Material

## References

[1] Pincus T Summey JA Soraci SA Jr. Wallston KA Hummon NP. Assessment of patient satisfaction in activities of daily living using a modified Stanford health assessment questionnaire. Arthritis Rheum. 1983;26:1346–53. 6639693 10.1002/art.1780261107

[2] Uhlig T Haavardsholm EA Kvien TK. Comparison of the Health Assessment Questionnaire (HAQ) and the modified HAQ (MHAQ) in patients with rheumatoid arthritis. Rheumatology (Oxford). 2006;45:454–8. 16287925 10.1093/rheumatology/kei181

[3] Matsuda Y Singh G Yamanaka H. Validation of a Japanese version of the Stanford health assessment questionnaire in 3,763 patients with rheumatoid arthritis. Arthritis Rheum. 2003;49:784–8. 14673964 10.1002/art.11465

[4] Tawaratsumida H Setoguchi T Arishima Y. Risk factors for bone loss in patients with rheumatoid arthritis treated with biologic disease-modifying anti-rheumatic drugs. BMC Res Notes. 2017;10:765. 29268799 10.1186/s13104-017-3086-7 PMC5740597

[5] Tokumoto H Tominaga H Arishima Y. Association between bone mineral density of femoral neck and geriatric nutritional risk index in rheumatoid arthritis patients treated with biological disease-modifying anti-rheumatic drugs. Nutrients. 2018;10:234. 29463015 10.3390/nu10020234 PMC5852810

[6] El Maghraoui A Rezqi A Mounach A Achemlal L Bezza A Ghozlani I. Prevalence and risk factors of vertebral fractures in women with rheumatoid arthritis using vertebral fracture assessment. Rheumatology (Oxford). 2010;49:1303–10. 20360038 10.1093/rheumatology/keq084

[7] Zerbini CAF Clark P Mendez-Sanchez L. IOF Chronic Inflammation and Bone Structure (CIBS) Working Group. Biologic therapies and bone loss in rheumatoid arthritis. Osteoporos Int. 2017;28:429–46. 27796445 10.1007/s00198-016-3769-2

[8] Singh JA Hossain A Tanjong Ghogomu E Mudano AS Tugwell P Wells GA. Biologic or tofacitinib monotherapy for rheumatoid arthritis in people with traditional disease-modifying anti-rheumatic drug (DMARD) failure: a Cochrane Systematic Review and network meta-analysis (NMA). Cochrane Database Syst Rev. 2016;2016:CD012437. 10.1002/14651858.CD012437 PMC6469573 27855242

[9] van der Heijde D Klareskog L Singh A. Patient reported outcomes in a trial of combination therapy with etanercept and methotrexate for rheumatoid arthritis: the TEMPO trial. Ann Rheum Dis. 2006;65:328–34. 16079172 10.1136/ard.2005.035709 PMC1798055

[10] Cawthon PM Blackwell TL Marshall LM. Osteoporotic Fractures in Men (MrOS) Research Group. Physical performance and radiographic and clinical vertebral fractures in older men. J Bone Miner Res. 2014;29:2101–8. 25042072 10.1002/jbmr.2239 PMC4335673

[11] Omata Y Hagiwara F Nishino J. Vertebral fractures affect functional status in postmenopausal rheumatoid arthritis patients. J Bone Miner Metab. 2014;32:725–31. 24362454 10.1007/s00774-013-0552-8

[12] Kvien TK Haugeberg G Uhlig T. Data driven attempt to create a clinical algorithm for identification of women with rheumatoid arthritis at high risk of osteoporosis. Ann Rheum Dis. 2000;59:805–11. 11005782 10.1136/ard.59.10.805 PMC1753011

[13] Dirven L van den Broek M van Groenendael JH. Prevalence of vertebral fractures in a disease activity steered cohort of patients with early active rheumatoid arthritis. BMC Musculoskelet Disord. 2012;13:125. 22824097 10.1186/1471-2474-13-125 PMC3485122

[14] Ghazi M Kolta S Briot K Fechtenbaum J Paternotte S Roux C. Prevalence of vertebral fractures in patients with rheumatoid arthritis: revisiting the role of glucocorticoids. Osteoporos Int. 2012;23:581–7. 21350894 10.1007/s00198-011-1584-3

[15] Takahashi K Setoguchi T Tawaratsumida H. Risk of low bone mineral density in patients with rheumatoid arthritis treated with biologics. BMC Musculoskelet Disord. 2015;16:269. 26420629 10.1186/s12891-015-0732-x PMC4589107

[16] Li P Schwarz EM O’Keefe RJ. Systemic tumor necrosis factor alpha mediates an increase in peripheral CD11bhigh osteoclast precursors in tumor necrosis factor alpha-transgenic mice. Arthritis Rheum. 2004;50:265–76. 14730625 10.1002/art.11419

[17] Shin A Park EH Dong YH. Comparative risk of osteoporotic fracture among patients with rheumatoid arthritis receiving TNF inhibitors versus other biologics: a cohort study. Osteoporos Int. 2020;31:2131–9. 32514765 10.1007/s00198-020-05488-9

[18] Mori Y Kuwahara Y Chiba S. Bone mineral density of postmenopausal women with rheumatoid arthritis depends on disease duration regardless of treatment. J Bone Miner Metab. 2017;35:52–7. 26369319 10.1007/s00774-015-0716-9

[19] Chen JF Hsu CY Yu SF. The impact of long-term biologics/target therapy on bone mineral density in rheumatoid arthritis: a propensity score-matched analysis. Rheumatology (Oxford). 2020;59:2471–80. 31984422 10.1093/rheumatology/kez655 PMC7449814

[20] Hong WJ Chen W Yeo KJ Huang P-H Chen D-Y Lan J-L. Increased risk of osteoporotic vertebral fracture in rheumatoid arthritis patients with new-onset cardiovascular diseases: a retrospective nationwide cohort study in Taiwan. Osteoporos Int. 2019;30:1617–25. 31127317 10.1007/s00198-019-04966-z

[21] Kato S Demura S Shinmura K. Associations between abdominal trunk muscle weakness and future osteoporotic vertebral fracture in middle-aged and older adult women: a three-year prospective longitudinal cohort study. J Clin Med. 2022;11:4868. 36013104 10.3390/jcm11164868 PMC9410457

