# Supplementary material for: Risk factors for vertebral fracture in rheumatoid arthritis patients using biological disease-modifying anti-rheumatic drugs (cases over 5 years): An observational study
Source: Medicine (Baltimore). 2024 Jul 5;103(27):e38740. doi: 10.1097/MD.0000000000038740 (PMC11224858; doi:10.1097/MD.0000000000038740)
Supplement: Supplementary file 1 [file medi-103-e38740-s001.docx]

Supplemental Table 1. Correlations between parameters of osteoporosis and disease duration.

| Parameter |  | Disease duration |
| --- | --- | --- |
| Lumbar spine BMD (g/cm^2^) | r | -0.14 |
|  | p | 0.11 |
| Lumbar spine YAM (%) | r | -0.13 |
|  | p | 0.15 |
| Femoral BMD (g/cm^2^) | r | -0.34* |
|  | p | <0.001 |
| Femoral YAM (%) | r | -0.31* |
|  | p | <0.001 |

BMD = bone mineral density, YAM = young adult mean.

*Statistically significant.
